# Rapid Immunomagnetic Negative Enrichment of Neutrophil Granulocytes from Murine Bone Marrow for Functional Studies *In Vitro* and *In Vivo*

**DOI:** 10.1371/journal.pone.0017314

**Published:** 2011-02-23

**Authors:** Mike Hasenberg, Anja Köhler, Susanne Bonifatius, Katrin Borucki, Monika Riek-Burchardt, Julia Achilles, Linda Männ, Kathleen Baumgart, Burkhart Schraven, Matthias Gunzer

**Affiliations:** 1 Institute for Molecular and Clinical Immunology, Otto-von-Guericke University Magdeburg, Magdeburg, Germany; 2 Institute of Clinical Chemistry and Pathobiochemistry, Otto-von-Guericke University Magdeburg, Magdeburg, Germany; 3 Leibniz Institute for Neurobiology, Research Group Neuropharmacology, Otto-von-Guericke University Magdeburg, Magdeburg, Germany; Ludwig-Maximilians-Universität München, Germany

## Abstract

Polymorphonuclear neutrophils (PMN) mediate early immunity to infection but can also cause host damage if their effector functions are not controlled. Their lack or dysfunction is associated with severe health problems and thus the analysis of PMN physiology is a central issue. One prerequisite for PMN analysis is the availability of purified cells from primary organs. While human PMN are easily isolated from peripheral blood, this approach is less suitable for mice due to limited availability of blood. Instead, bone marrow (BM) is an easily available reservoir of murine PMN, but methods to obtain pure cells from BM are limited. We have developed a novel protocol allowing the isolation of highly pure untouched PMN from murine BM by negative immunomagnetic isolation using a complex antibody cocktail. The protocol is simple and fast (∼1 h), has a high yield (5–10*10^6^ PMN per animal) and provides a purity of cells equivalent to positive selection (>80%). Most importantly, cells obtained by this method are non-activated and remain fully functional *in vitro* or after adoptive transfer into recipient animals. This method should thus greatly facilitate the study of primary murine PMN *in vitro* and *in vivo*.

## Introduction

Polymorphonuclear neutrophils (PMN) are the most abundant and, arguably, most important cell type of the innate immune system. By non specific mechanisms such as phagocytosis and extracellular killing via toxic agents they mediate the first line of defense against infection. This early pathogen control provides the relatively slow adaptive immune system with a time window allowing to develop specifically tailored responses that can ultimately sterilize the body from the infection and provide long-term protection [1]. However, despite many decades of research still new principal defense mechanisms of PMN are being uncovered such as the formation of extracellular DNA-nets [2], [3]. Furthermore, the cells are also increasingly being recognized as mediators of host destruction by uncontrolled activity, e.g. in the pathogenesis of rheumatoid arthritis [4] or during viral infection [5]. Also the function of PMN during mobilization by cytokines such as G-CSF, which is routinely used in the clinic since many years, is still not fully understood [6]. Thus, PMN physiology remains a central and actively investigated topic of immunological and hematological research.

To analyze the function of primary PMN it is necessary to enrich these cells directly from source organs to a high purity. In humans the cells are easily obtained by density gradients from peripheral blood, which is rich in circulating PMN and available in sufficient quantities from volunteers or patients [7]. However, many experimental strategies, especially those dealing with infection defense or hematologic mobilization, work in suitable animal systems, mainly based on mice. Isolation of PMN from mice is much more difficult than from humans. Peripheral blood is not an ideal source, as the volume that can be obtained from a single animal (routinely less than 1 ml) is very low. In addition, as opposed to human blood, where up to 60% of circulating leukocytes are PMN this value reaches only 6–25% in the blood of normal mice with a total number of 1–1.5*10^6^ cells per ml [8]. A much more convenient source of murine PMN is, therefore, bone marrow (BM), which is easily obtained by simple flushing the large long bones of the hind legs and contains 20–40% of mature PMN with a total number of ∼5*10^7^ cells [6]. However, the methods to enrich PMN from BM to a reasonable purity that allows unbiased study are still limited.

In principle two methods for the enrichment of cells can be distinguished, positive or negative selection. Positive selection makes use of a known specific target structure on the cell type of interest that can be labeled, typically with a specific antibody, and then used for isolation either by flow cytometry or immunomagnetic separation. Although these approaches are easy, rapid and reach very high purities of the wanted cells, they suffer from the principal drawback that the labeling agents remain bound to the cells and can then severely alter their function *in vitro* and *in vivo* [9]. Therefore, negative selection, where all non-wanted cells are labeled and removed from a mixture is much more advantageous. Previously, several protocols for the negative selection of murine PMN have been published. One type relies on peripheral blood as source, but suffers from low yield [8] or may in addition require injection of heparin into donor animals that is likely to influence cellular function [10]. The total duration of 3 h for the latter protocol is also not well suited to study a cell type with an average *in vivo* half life of 6 hours [11]. A different protocol used BM as source, but employed technically difficult separation via discontinuous percoll gradients to obtain the cells [12]. While all of these methods reach their goal, they nevertheless are hampered by technical difficulties that might have precluded their wide spread use.

Optimized negative immunomagnetic isolation protocols of “untouched cells” directly from suitable target organs are characterized by ease of use, good yield, excellent purity, rapidity and a gentle treatment of the cells during the procedure [9]. Thus, they have become a gold-standard for the study of primary immune cells. Such a protocol has so far been lacking for the isolation of murine PMN. We present here a novel antibody cocktail that allows the enrichment of untouched PMN from murine BM to high purity. In contrast to positively selected cells, untouched PMN are non-activated and retain full functionality *in vitro*. Most importantly and in contrast to positively selected cells they can be adoptively transferred to recipient animals and behave similar to endogenous cells in a model of LPS peritonitis. The combination of our novel isolation protocol with the adoptive transfer model should thus greatly facilitate the functional analysis of PMN in clinically relevant *in vivo* models in the future.

## Results

### Identification of an antibody cocktail for the purification of murine neutrophils from bone marrow

**Figure 1:**
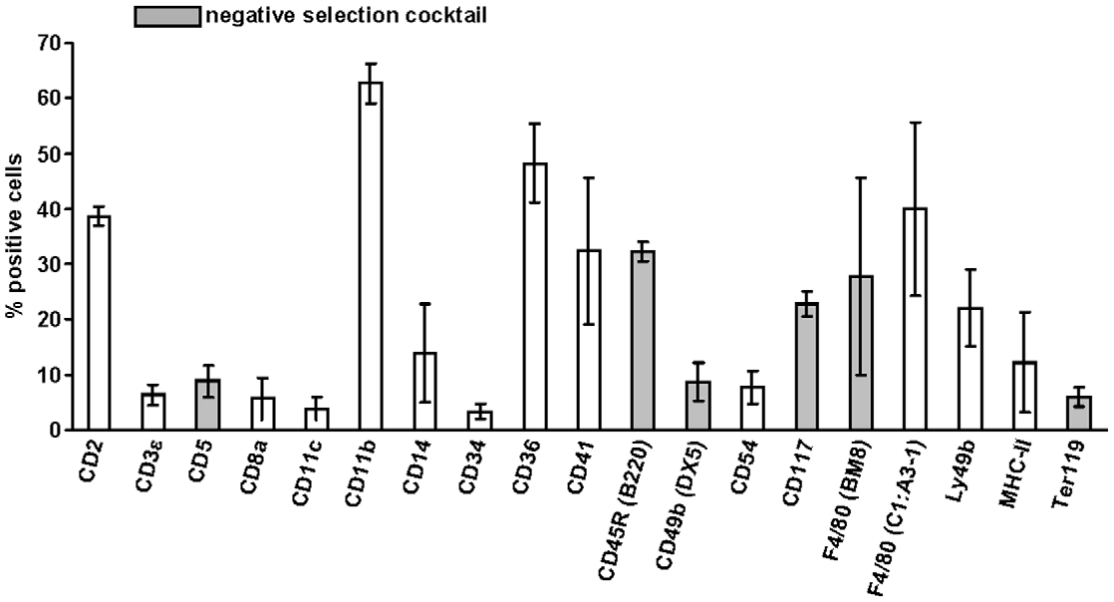
Cell composition of murine BM. The bone marrow of 8–10 weeks old female C57BL/6 mice was isolated and after erythrocyte lysis the percentage of the particular surface marker positive cells on total living cells was assessed. Gray bars indicate the antibodies which were used in the neutrophil-isolation cocktail presented in this study. Percentages are means of at least three independent experiments.

To establish a protocol that would allow the negative isolation of murine neutrophils from bone marrow we first had to identify good candidate surface markers which are present on marrow resident cells but absent from neutrophils. It was essential, that all markers were expressed on the surface of cells since we wanted to isolate living cells without the need for fixation and permeabilization. To this end we used a selection of fluorescently labeled monoclonal antibodies binding to a wide spectrum of different surface markers and measured the amount of antibody binding cells in normal bone marrow by flow cytometry. (Fig. 1) shows the relative percentage of cells and the identities of the labeled antigens for which we could detect binding of specific antibodies in normal murine bone marrow. The next step required to identify those antigens that were absent on neutrophils and, at the same time, non-overlapping with each other in terms of cellular specificity. Thus, e.g. CD14 was not a good marker, as it was 100% overlapping with F4/80, while F4/80 stained also other cells that were not detected by CD14. A cocktail containing anti-CD14 antibodies would thus not yield as pure neutrophils as one containing F4/80. Specifically with F4/80 we also found, that the antibody clone C1:A3-1 did not yield good results, while clone BM8 was very effective. Finally, all potential candidates were checked in double stainings against the neutrophil-specific marker Gr-1 stained by the antibody RB6-8C5 [13], [14] and were only taken, if Gr-1 positive cells were not stained by a defined antibody. With these selection criteria we finally identified a cocktail consisting of antibodies specific for 6 different antigens, namely CD5 (T cells), CD45R/B220 (B cells), CD49b/DX5 (NK cells), CD117 (Mast cells and hematopoietic stem cells), F4/80 (clone BM8, macrophages) and Ter119 (erythrocytes). A mixture of antibodies against these 6 types of cells/antigens was then labeled with biotin. Bone marrow harvested by flushing of femoral bones was freed of erythrocytes by short-term hypotonic lysis as described [15] and then incubated with the biotinylated antibody mixture. The optimized amount of each antibody in the cocktail used for the number of bone marrow cells in a sample is given in table 1.

**Table 1:** Antibodies used for isolation of neutrophils.

Target	Clone	Isotype	Company	Stock Concentration	ng Antibody per10^7^ cells
CD5	53-7.3	Rat IgG2a, κ	BD Biosciences	0.5 mg/ml	250
CD45R	RA3-6B2	Rat IgG2a, κ	BD Biosciences	0.5 mg/ml	125
CD49b	HMa2	Armenian Hamster IgG	eBioscience	0.5 mg/ml	250
ckit/CD117	2B8	Rat IgG2b, κ	eBioscience	0.5 mg/ml	100
F4/80	BM8	Rat IgG2a, κ	eBioscience	0.5 mg/ml	500
TER119	TER 119	Rat IgG2b, κ	BioLegend	0.5 mg/ml	100

The shown amount of each antibody, which was the minimal quantity necessary for optimal depletion of labeled cells, was titered for each antibody individually at saturating doses of magnetic beads. After MACS purification with varying AB concentrations, samples were tested by flow cytometry for the degree of cell depletion, and the minimal dose of antibody giving optimal depletion was used.

After incubation the unbound antibodies were washed away from the cells which was a critical step to avoid binding of free antibodies to magnetic particles used in the next step of the protocol. The cells were then incubated with magnetic beads (MACS, Miltenyi Biotec, Germany) labeled with the biotin binding molecule streptavidin. Then, bead coupled cells were removed from the mixture by immunomagnetic separation following the manufacturer's recommendation leaving behind highly purified neutrophils in the supernatant. After a final washing step the cells were ready for use. The whole procedure from the bone marrow harvest to finally purified cells can be done within 1-1.5 hours and does not require technically challenging steps or exposure of the cells to potentially noxious substances other than a short incubation in hypotonic erythrocyte lysis buffer.

### Quality comparison of the novel negative to the established positive selection

**Figure 2:**
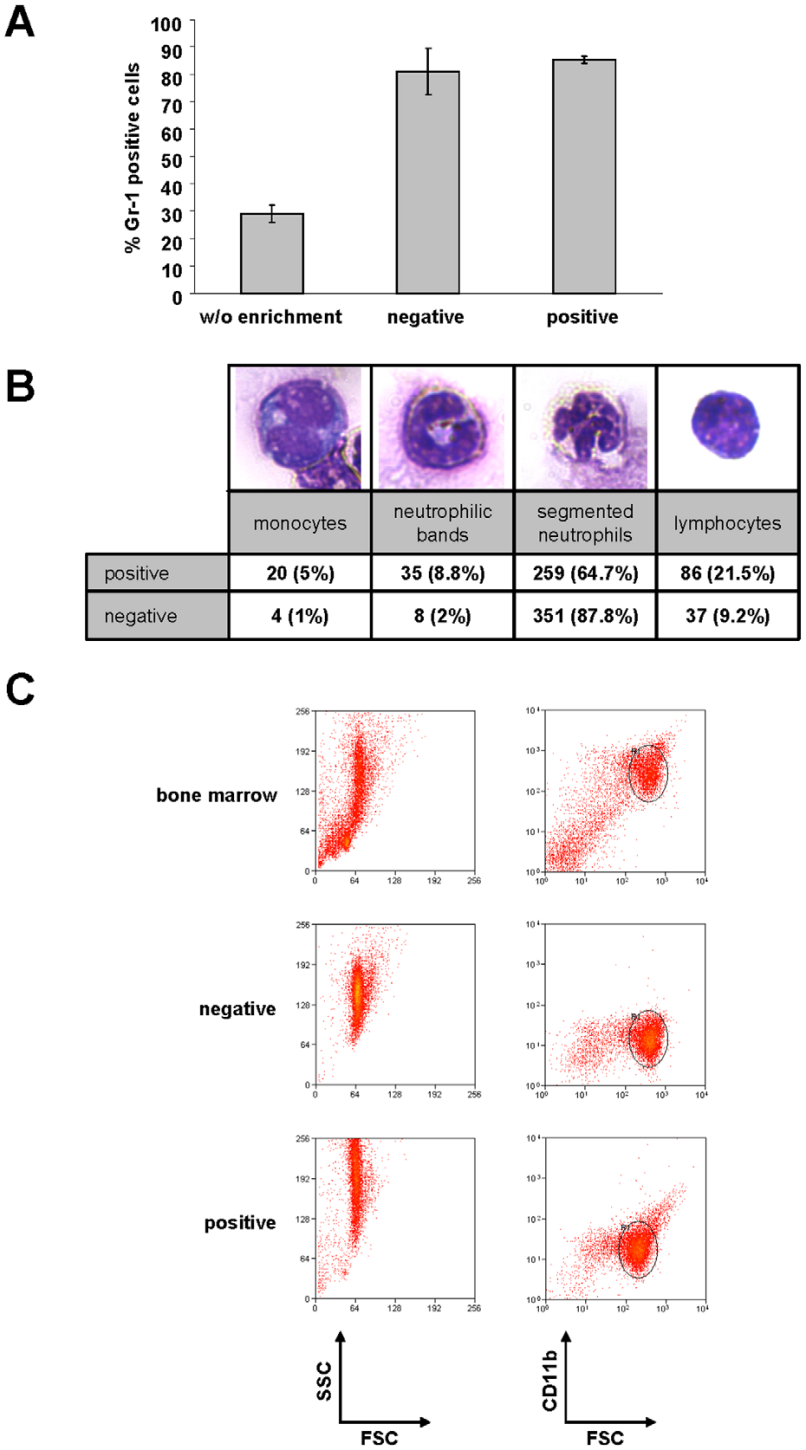
Neutrophil purity. (a) Neutrophil granulocytes were enriched by either negative depletion, positive selection or by PBS treatment following the negative depletion protocol ( =  w/o enrichment) and cell purity was assessed by estimating the percentage of Gr-1^+^/CD11b^+^ cells on the total cell number. Values are means of three independent experiments. (b) After neutrophil preparation following the positive or negative isolation protocol the cells were spread on a microscope glass slide using a cytocentrifuge and subsequently total cell type numbers were counted following a Pappenheim staining. Numbers are total counts of two independent cell preparations. The photographs show representative images of cells scored for the relevant class. (c) Representative flow cytometry plots of cells isolated with the different protocols. Please note the high side scatter cloud of neutrophils after positive selection.

Having established the principal antibody cocktail for negative isolation we had to compare the performance of this cocktail with the established positive isolation using Gr-1 as a neutrophil specific marker. The purity of the cells was almost identical with both methods based on the analysis of the combined expression of Gr-1 and CD11b on the isolated cells (Fig. 2A and table 2). However, an analysis of the sorted cells by microscopic inspection using Pappenheim-stained cytocentrifuge preparations identified, that the Gr-1-mediated positive isolation yielded a considerable contamination of the cells with monocytic cells, neutrophilic precursors and an unidentified lymphocytic subtype (Fig. 2B), while the negatively isolated cells were almost 90% mature neutrophils with the major contamination being of lymphocytic morphology. This suggests, that positive sorting for Gr-1 also isolates a substantial fraction of cells expressing lower amounts of the marker, which are known to contain neutrophil precursors and monocytic cells [14] while our novel negative sorting protocol specifically enriches Gr-1^high^ mature neutrophils. The presence of ∼5% lymphocytic cells in the Gr-1^high^ fraction has been described earlier and the identity of the cells could so far not be clarified. Although a population of CD8 memory T cells has been described to express Gr-1 [16], this is unlikely to be the contaminant, as CD5 is expressed on all mature T cells including memory cells, thus these cells should have been depleted with the current cocktail. Furthermore, morphologically the cells appear to be small lymphocytes (Fig. 2B).

**Table 2:** Cell numbers during different steps of the purification process.

Step	Cells per mouse (×10^6^)		Purity (% Gr-1^+^/CD11b^+^ of all cells)	
	positive	negative	positive	negative
Erythrocyte Lysis	57.6±1.34		29.09±3.21	
MACS	4.68±1.47	6.7±2.14	85.33±1.39	81.09±8.5

Numbers are mean values of at least four independent experiments.

A further analysis of unfractionated bone marrow and isolated cells showed that the cells purified by positive enrichment showed a large increase in side scatter indicative of increased granular content and thus cellular activation, while the flow cytometric morphology of negatively isolated cells remained unchanged relative to the appearance in the bone marrow (Fig. 2C). Collectively these data show that the newly developed antibody cocktail is able to isolate highly pure mature neutrophils which remain in a resting state during the protocol.

### Functional studies *in vitro*

**Figure 3:**
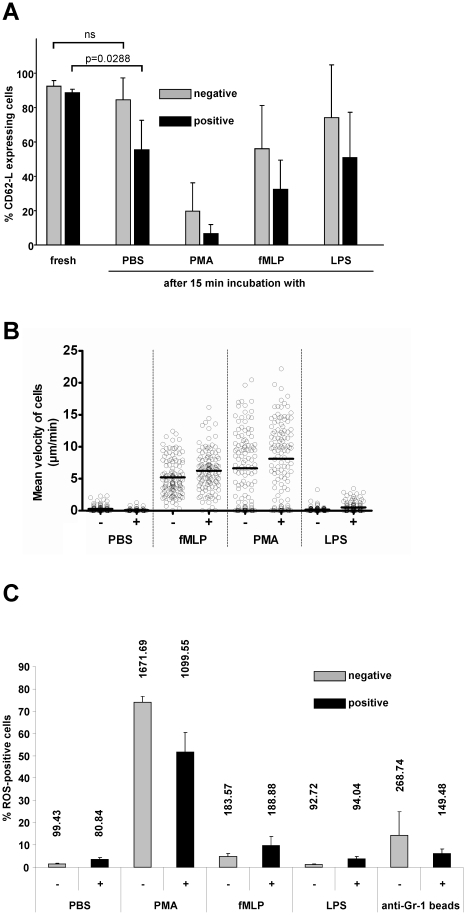
Neutrophil characterization. (a) After neutrophil preparation following the positive or negative isolation protocol the cells were treated with either PBS, PMA, fMLP or LPS for 15 minutes and the change of positive cells for CD62L in % on total living cells was estimated relative to freshly isolated, untreated cells by flow cytometry. Values are means of three independent experiments. (b) Positively (+) or negatively (−) isolated neutrophils were observed by time-lapse video microscopy in the presence of either PBS, PMA, fMLP or LPS for 3 h and cell velocity was assessed using a cell tracking software module. A total of 120 cells in 3 independent experiments per condition were analyzed. Black bars indicate the mean velocity values. (c) Right after positive (black bars) or negative isolation (grey bars) neutrophils were treated with PBS, PMA, fMLP or LPS for 15 min, subsequently stained with the ROS indicator DCFH and analyzed by flow cytometry for the occurrence of green  =  ROS positive cells. The mean fluorescence intensity (MFI) values for the measurements are stated above the particular bars. All values are means of four independent experiments except for the positively isolated and PBS treated population for which three independent experiments were analyzed.

Next we asked, whether the neutrophils isolated with the novel protocol were functionally mature neutrophils. To study this we investigated three key cellular processes of neutrophils. First we tested the expression of CD62-L, a surface marker of non-activated neutrophils, that can be quickly shedded following activation [17], [18]. While both, positively and negatively isolated cells showed equally high levels of CD62-L directly after isolation, a 15 min. incubation period in PBS already led to a significant reduction of the marker in positively as opposed to negatively selected cells (Fig. 3A). Triggering with LPS did not increase this shedding activity however the treatment of the cells with the pro-migratory tripeptide fMLP or the phorbolester PMA strongly induced CD62-L shedding in these cells (Fig. 3A) as expected. These data suggested that the positive isolation method induced some degree of pre-activation in isolated neutrophils, which was not present in the negatively isolated cells. However, both types of isolation yielded cells with the principal ability to function with regard to CD62-L shedding. The cell surface expression analysis of classical activation markers (CD18 (β-chain of the β2-integrins), CD54 (ICAM-1) and CD88 (C5a receptor)) revealed an identical expression profile for neutrophils of both isolation protocols upon stimulation with PBS, PMA, fMLP and PBS (data not shown).

Next we investigated the migration of neutrophils *in vitro*. To this end isolated cells were incubated in tracking chambers in the presence of various stimuli and cell migration was analyzed by time-lapse video microscopy followed by computer-assisted single cell tracking as described [19]. While non- or LPS-stimulated cells did not show profound migration, the presence of PMA or fMLP led to massively increased motility of the cells as observed earlier [7] (Fig. 3B).

Finally, we investigated the ability of neutrophils to produce reactive oxygen species (ROS). This is an important defense mechanism of neutrophils [20] and thus a functional ROS system is a measure of neutrophil fitness. We found a high degree of variability in ROS positive cells within negatively isolated neutrophils, which was further increased and homogenized over almost the entire population by the addition of either PMA or fMLP, while LPS did not increase the level of ROS (Fig. 3C). Interestingly, positively selected neutrophils showed a much lower level of spontaneous ROS production which could not be increased by fMLP or LPS, but only by PMA. These data suggested that negatively selected neutrophils presented with a varying degree of baseline ROS activity which, however, was sensitive to specific positive triggering, while positively selected cells could only be activated by the supra-agonistic trigger PMA.

### Functional studies *in vivo*

**Figure 4:**
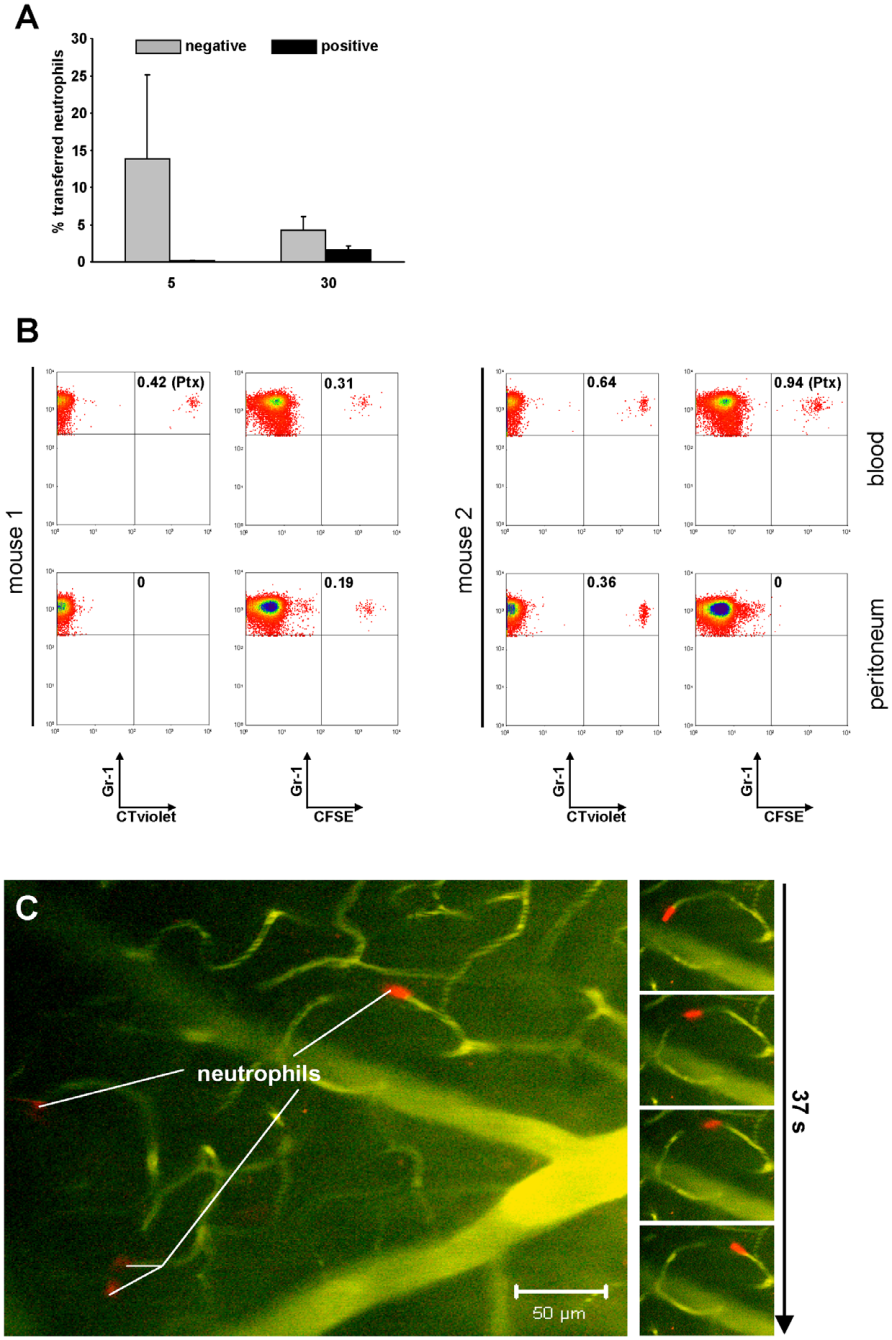
Application examples using adoptive cell transfer of isolated neutrophils. (a) Positively or negatively isolated neutrophils from Lys-EGFP mice were adoptively transferred (1×10^7^ cells i.v.) into 8–10 weeks old, female C57BL/6 animals. 5 and 30 minutes after injection blood samples of the recipients were analyzed for the number of green donor neutrophils by flow cytometry. Values are means of three independent experiments. (b) Neutrophil recruitment in a murine peritonitis model. After negative isolation PMN were incubated in PBS or 200 ng/ml Pertussis toxin for 60 minutes before washing, CTCFSE- or CTViolet-staining and adoptive transfer into wild type recipients. 15 minutes later animals were injected i.p. with 15 ng LPS in 150 µl PBS. 2 h later animals were bled (75 µl), then sacrificed, their peritoneum was flushed with 5 ml PBS and cells contained within the wash were analyzed for the presence of Gr-1 as well as CTCFSE- or CTViolet-positive cells by flow cytometry. Please note that pertussis-treated cells remain in the blood of mice, while PBS-treated cells effectively extravasate into the peritoneum. A change in the used dyes (mouse 2) did yield the same result. (c and Movie S1) Intravital 2-photon brain microscopy of a C57BL/6 animal that had received negatively isolated, CTO stained neutrophils as well as FITC-dextran i.v. shortly before onset of microscopy. The big image shows several red neutrophils in a green blood vessel. The series of images depicts a single neutrophil squeezing through a capillary within 37 sec. of observation. The cells can also be observed in Movie S1.

Ultimately, studies on cellular function should be performed in the natural environment rather than in a test tube. Thus we wished to investigate, whether neutrophils negatively selected by our novel antibody cocktail would still function after adoptive transfer into recipient animals. To this end isolated cells were stained with fluorescent dyes (CellTracerCFSE, CellTrackerOrange or CellTracerViolet), adoptively transferred into wild type recipients and then tested by various assays. First we directly compared the ability of positively and negatively isolated cells to generate a transient circulatory chimerism in the host animals. To this end peripheral blood of the recipient animals was analyzed for the presence of transferred cells 5 or 30 minutes after adoptive transfer (Fig. 4A). The analysis showed that positively selected cells were hardly detectable at 5 and only to a low extent at 30 minutes after transfer, while negatively selected cells were clearly detectable at both time points with a decreasing concentration probably reflecting the normal cellular turnover (Fig. 4A).

Next we asked whether the cells were still able to perform physiological cellular functions *in vivo.* A powerful way to recruit neutrophils rapidly to a site of inflammation is LPS-mediated peritonitis. Here the local inflammation leads to a massive flooding of neutrophils into an otherwise almost neutrophil free environment. This recruitment is mediated by the chemokine receptor CXCR2 on neutrophils which detects the chemokines KC and MIP-2 expressed in the inflamed peritoneum [21], [22]. Chemokine receptors function via G-protein coupled signaling [23] which can be blocked be treatment of cells with pertussis toxin [24]. Thus we reasoned that adoptively transferred neutrophils should be able to enter an LPS-inflamed peritoneum in a host animal, while this would not work, when the cells had been pretreated with pertussis toxin. To test this hypothesis, we negatively isolated neutrophils with our novel cocktail, treated one half of the preparation for 60 minutes with pertussis toxin, while the other half remained untreated. The cells were then stained either with CFSE or CellTracerViolet and adoptively transferred into mice. 15 minutes later we injected LPS *i.p.* and measured the degree of blood chimerism as well as the presence of endogenous and adoptively transferred neutrophils in the peritoneum of the same mice by flow cytometry 2 hours later. The results clearly showed, that both, normal and pertussis toxin-treated cells were detectable in the circulation of the recipient animals, while only untreated cells were also able to migrate into the LPS-inflamed peritoneum. This was not an effect of the staining procedure, as a reversal of the dyes had no influence on the observed phenomenon (Fig. 4B). Thus, negatively isolated neutrophils retain normal functional activity *in vivo*.

Finally we wished to test, whether the adoptively transferred cells were also detectable by direct microscopic observation *in vivo*. To this end we performed intravital 2-photon microscopy [15], [25] on cranial blood vessels of anesthetized animals that had received a transfer of negatively isolated neutrophils stained with CellTrackerOrange, thus glowing red. Indeed we could observe individual red cells rushing through blood vessels or squeezing themselves through tight capillaries thereby showing autonomous migration (Fig. 4C and Movie S1). Of note, initial attempts to observe positively isolated neutrophils after adoptive transfer were entirely unsuccessful as we could never detect a single circulating cell. This further explains our inability to detect positively selected cells in the circulation of mice by flow cytometry (Fig. 4A) and suggests that positively selected neutrophils do not function properly *in vivo*.

Collectively these data show, that our novel protocol for negative isolation yields fully functional neutrophils that can be used for a wide variety of *in vitro* or *in vivo* functional studies.

## Discussion

The negative isolation protocol shown here combines several advantages that make it particularly useful for the analysis of murine models. (1) Instead of using peripheral blood it exploits the BM as source for the cells. As opposed to peripheral blood, BM is easily obtained in reproducible quantities and contains much greater numbers of PMN. Importantly, the majority of neutrophils in this tissue is of the Gr-1^high^ phenotype and represents mature neutrophils displaying the same expression profile of classical activation markers upon stimulation as cells isolated by positive selection. The finding of our cytosoin preparations, that even more mature, segemented PMNs could be isolated by negative than by positive selection was quite remarkable as the BM is the place of neutrophil development. However, although cells prepared with our protocol can function like peripheral blood counterparts in adoptive transfer models, as shown in this study, one should be aware that cells already circulating in the peripheral blood are likely to be more mature due to the fact, that they have already responded to a functional mobilization trigger such as G-CSF [26]. Future experiments need to investigate, how strong this difference is. (2) The protocol provides untouched and non-activated cells. Indeed, the fact that the specific binding of an antibody to the Gr-1 molecule of PMN induces their activation is corroborated by our finding of rapid partial CD62-L loss in cells positively selected via Gr-1, while negatively selected cells remained CD62-L^high^.during *in vitro* culture Nevertheless, activation of the cells via a variety of proinflammatory stimuli led to rapid loss of CD62-L proving the ability of the negatively isolated cells to respond to physiological stimuli. (3) Most importantly, in contrast to positively selected cells, the untouched PMN did function normally after adoptive transfer *in vivo*. We were never able to detect significant numbers of circulating PMN, which had been adoptively transferred following positive selection via Gr-1, while untouched cells remained in the circulation for extended periods. Given the fact that Gr-1 antibodies are extremely efficient to mediate long-term neutropenia in mice [8], [27] one can speculate that the antibodies binding to the positively selected cells also mediated the rapid destruction of the transferred PMN, e.g. by complement mediated lysis in the host [28]. An alternative possibility is that the pre-activated positively selected cells were binding to endothelia in the recipient animals and thus were not available in the free circulation [29].

In any case, the novel isolation protocol described here provides an easy way to obtain sufficient quantities of mature and highly pure primary PMN from the mouse. While this can be used for an array of *in vitro* functional tests such as phagocytosis [30], NET-formation [3] and migration [31], the most promising approach is clearly the adoptive transfer. In the way demonstrated here, the analysis of peripheral blood by multicolor FACS first allows to verify the degree of chimerism in each recipient. Functional assays then allow to directly test the functional abilities of the transferred cells relative to the internal control of host cells, a very powerful approach. We demonstrate here the capability of wild type-like transferred cells to function like wild type cells. At the same time the role for G-protein coupled signaling for PMN function *in vivo* can be demonstrated here in a model resembling a true clinical problem. Given the ease of this novel approach, it should be possible in the future, to test many known genetic mutations or well defined inhibitors, e.g. of signaling pathways or key enzyme systems, for their impact on central PMN functions. This is not limited to LPS peritonitis as a model. We have demonstrated transferred cells rolling on brain blood vessels after local inflammation and a model of acute lung inflammation leading to NET formation [3] should also be possible to be investigated for critical PMN functions with this approach. Any other model requiring neutrophil function should also benefit from this novel technical approach.

Thus, in summary, our novel immunomagnetic negative isolation protocol for PMN alone or together with the adoptive transfer approach should allow the comprehensive study of murine PMN physiology *in vitro* and *in vivo* and thus considerably boost this important field of research in the future.

## Materials and Methods

### Ethics statement

All animal experiments were in compliance with the German animal protection law in a protocol approved by the Landesverwaltungsamt Sachsen-Anhalt (file number: 203.h-42502-2-881 University of Magdeburg).

### Animals

In all experiments female mice between 10 to 14 weeks of age were used. C57BL/6 animals were purchased from Harlan Winkelmann (Borchen, Germany) and Lys-EGFP mice (transgenic C57BL/6 mice expressing EGFP under the neutrophil- and macrophage-specific lysozyme M promotor) [32] were bred at the animal facility of the Otto-von-Guericke University Magdeburg. All mice were housed under SPF conditions until start of the particular experiment.

### Media and Supplements

**PBS:** NaCl [136.9 mM], KCl [2.7 mM], Na_2_HPO_4_ [8.1 mM], K_2_HPO_4_ [1.47 mM]; **Erythrocyte Lysis Buffer (ELB):** NH_4_Cl [0.15 mM], KHCO_3_ [1 mM], Na-EDTA [0.1 mM]; **MACS buffer:** PBS supplemented with Fetal Calf Serum (FCS; PAN Biotech; Aidenbach, Germany) [2% v/v], EDTA [2 mM]; [**Complete Medium (CM):** RPMI 1640 (Biochrom; Berlin, Germany) supplemented with β-mercaptoethanol (Gibco/Invitrogen; Darmstadt, Germany) [50 µM], FCS [10% v/v], HEPES (Gibco/Invitrogen) [10 mM], L-glutamine (Biochrom) [2 mM], Na-pyruvate (PAA; Pasching, Austria) [1 mM], Non Essential Amino Acids (NEAA; Gibco) [1×], Pen/Strep (Gibco) [100 U/ml].

### Antibodies

In all experiments where the BM composition was defined, following ABs were used for FACS analysis (bold and italic: antibodies which have been subsequently used in the AB cocktail for neutrophil isolation): anti-CD2-Biotin (Biolegend; San Diego, USA; Clone: RM2-5), anti-CD3ε-APC (eBioscience; San Diego, USA; Clone: 145-2C11), ***anti-CD5-Biotin (BD Biosciences; Heidelberg, Germany; Clone: 53-7.3)***, anti-CD8a-Biotin (BD Biosciences; Clone: 53-6.7), anti-CD11c-APC (Biolegend; Clone: N418), anti-CD11c-PE (eBioscience; Clone: N418), anti-CD11b-PerCP (Biolegend; Clone: M1/70), anti-CD14-Biotin (eBioscience; Clone: Sa2-8), anti-CD34-Biotin (Biolegend; Clone MEC14.7), anti-CD36-Biotin (Biolegend; Clone: HM36), anti-CD41-Biotin (eBioscience; Clone: 1BioMWReg30), anti***-CD45R(B220)-Biotin (BD Biosciences; Clone: RA3-6B2)***, ***anti-CD49b(DX5)-Biotin (eBioscience; Clone: HMa2)***, anti-CD54-Biotin (Biolegend; Clone: YN1/1.7.4), ***anti-CD117-Biotin (eBioscience; Clone: 2B8)***, ***anti-F4/80-Biotin (eBioscience; Clone: BM8)***, anti-F4/80-Biotin (Biolegend; Clone: C1:A3-1), anti-Ly49b-Biotin (eBioscience; Clone: 14B11), anti-MHCII-FITC (eBioscience; Clone: NIMR-4), ***anti-Ter119-Biotin (Biolegend; Clone: TER119)***. All biotinylated ABs were detected with Streptavidin-APC (BD Biosciences).

In order to assess the amount of neutrophils and macrophages in a cell population by FACS analysis, directly fluorophor-coupled detection ABs were used: anti-Gr-1(Ly6G)-PE (BD Biosciences; Clone: RB6-8C5), anti-Gr-1(Ly6G)-APC (BD Biosciences; Clone: RB6-8C5), anti-F4/80-FITC (Serotec; Clone: C1:A3-1), anti-CD11b-APC (Biolegend; Clone: M1/70). The expression of neutrophilic surface markers was evaluated in FACS experiments by use of these ABs: anti-CD18-PE (Biolegend; Clone: M18/2), anti-CD54-PE (Biolegend; Clone: YN1/1.7.4), anti-CD62L-PE (Biolegend; Clone: MEL-14), anti-CD88-PE (Acris; Clone: C5R1).

### Bone marrow characterization

Immediately after cervical dislocation one hind limb of a C57Bl/6 mouse was prepared and the whole BM was flushed out of both, tibia and femur, by use of a 21-gauge needle attached to a 10 ml syringe filled with PBS. After centrifugation (300× g/5 min/room temperature (RT)) the supernatant was removed and erythrocytes were eliminated by a 1 min incubation step in 5 ml ELB. Subsequently the resulting cells were washed with 10 ml PBS and divided onto a 96-Well cell culture plate with 10^6^ cells per well. After another centrifugation step (300× g/5 min/RT) the cells were directly dispersed in 70 µl of the particular AB solution and incubated for 20 min at RT in the dark. Then the cells were washed once with 200 µl PBS and resuspended for FACS analysis in a total volume of 400 µl buffer. The amount of positive cells for the respective marker was then determined in comparison to the appropriate AB isotype control.

### Isolation of neutrophils by negative depletion

Both hind limbs of a mouse were removed directly after cervical dislocation and a single cell suspension w/o erythrocytes was generated as mentioned above. After centrifugation (300× g/5 min/RT) the cells were then resuspended in an appropriate volume of antibody cocktail (see Table 1 for details) and the suspension was constantly turned around in a rotating shaker at 4°C for 10 min. By a centrifugation step (300× g/5 min/RT) the supernatant was removed and the cell pellet was suspended in 100 µl MACS Buffer per 10^7^ total cells. Right before the suspension was then incubated on the rotating shaker for another 15 min, 15 µl of “Anti-Biotin-Microbeads” (Miltenyi Biotec, Bergisch Gladbach, Germany) per 10^7^ total cells were added. Subsequently the cells were washed with 10 ml MACS buffer (300× g/5 min/RT), taken up in 500 µl of this solution and filled into a pre-equilibrated “LS MACS column” (Miltenyi Biotec). After liquid flow has stopped the column was washed three times with 1 ml and finally once with 6 ml MACS buffer respectively and the whole flow-through was collected. The obtained cells were then washed once with 10 ml PBS and in a closing step the neutrophil cell number was assessed.

### Isolation of neutrophils by positive isolation

Positive isolation of neutrophils was achieved by “anti-Ly6G and Ly6C magnetic particles” in combination with the related cell separation system from BD Biosciences following manufacturer's instructions, including the “Mouse BD Fc Block™” reagent.

### Determination of neutrophil purity

After either positive isolation, negative depletion or w/o enrichment (i.e. total cells after erythrocyte lysis) of neutrophil granulocytes the cells were stained with an anti-CD11b/anti-Gr-1 AB mixture as mentioned above and analyzed for double positive cells by flow cytometry.

### Leukocyte differentiation

Neutrophil granulocytes were isolated by either positive or negative enrichment. 8*10^4^ cells were then transferred onto a microscopic glass slide by cytospin centrifugation (1400 rpm). After the samples had subsequently been air-dried the cells were fixed and labeled by a Pappenheim's stain. Briefly, cells were incubated 4 min in “May-Grünwalds Eosin-Methylenblaulösung” (Merck, Darmstadt, Germany), washed once with distilled water for 1 min, stained with diluted “Giemsa A zur-Eosin-Methylenblaulösung” [1:11.5 in H_2_O] (Merck) and finally rinsed again with distilled water for another minute. In each sample 100 cells were analyzed for total numbers of neutrophilic bands, segmented neutrophils, leukocytes or macrophages using a 100× oil immersion objective on an upright BH-2 transmission light microscope (Olympus, Hamburg, Germany).

### Determination of surface marker expression

Straight after positive or negative enrichment of neutrophils 5*10^6^ cells were resuspended in 5 ml CM respectively and stimulated with either PBS [10 µl/ml], **phorbol 12-myristate 13-acetate (PMA; Sigma-Aldrich; Hamburg, Germany) [10 ng/ml],** formyl-met-leu-phe (fMLP, Sigma-Aldrich) [0.1 mM] or lipopolysaccharide (LPS, Sigma-Aldrich) [2.5 ng/ml] at 37°C. After 15 min the cells were immediately cooled down to 4°C, washed twice with 10 ml PBS, stained for CD18, CD54, CD62L and CD88 expression and analyzed by flow cytometry.

### Determination of cell velocity

2*10^6^ of positively or negatively enriched neutrophils were resuspended in 150 µl CM supplemented with either PBS [10 µl/ml], PMA [10 ng/ml], fMLP [0.1 mM] or LPS [2.5 ng/ml] and transferred to microscopic “Press-To-Seal™” silicon chambers (2 cm ×0.5 mm; Invitrogen). Straight after sealing with a cover slip time-lapse video microscopy was started at 1 frame per min for 3 hours using a 60× LUMPLFL W/IR (NA 0.9) lens in a cell∧R fluorescence widefield microscopy system (Olympus). Cell migration of 20 cells per movie was analyzed subsequently by computer-assisted cell tracking using a software application developed for this study as described [33].

### Determination of ROS production

Following either positive or negative isolation of neutrophils 1*10^6^ cells were resuspended in 1 ml CM and stimulated with either PBS [10 µl/ml], **PMA [10 ng/ml]**, fMLP [0.1 mM] or LPS [2.5 ng/ml] at 37°C for 15 min. Then the cells were immediately cooled down to 4°C, washed once with 1 ml PBS and resuspended in 500 µl ROS detection solution (CM-H2DCFDA (Invitrogen) [1 µM in PBS]) supplemented with an anti-Gr-1-APC antibody to simultaneously stain the neutrophil population. After 20 min incubation at RT the cells were washed with 1 ml PBS and after centrifugation (300× g, 5 min, RT) the sedimented cells were dispersed in 200 µl PBS and analyzed by flow cytometry for FL-1/FL-4 double positive events.

### Neutrophil intravital survival experiment

2*10^7^ positively or negatively enriched neutrophils of Lys-EGFP mice were adoptively transferred into a naïve C57BL/6 recipient. 5 and 30 min after i.v. injection 75 µl peripheral blood were drawn and analyzed for cell numbers of green fluorescent Gr-1^+^ neutrophils by flow cytometry following 2 steps of erythrocyte lysis.

### Intravital 2-photon imaging

Right after isolation of neutrophils from C57Bl/6 mice the cells were stained with CellTrackerTM Orange (CTO; Invitrogen) by an incubation of 3*10^7^ cells in 1 ml CTO staining solution [10 µM in PBS] for 30 min at 37°C. After one washing step with 10 ml PBS 2*10^7^ neutrophils were resuspended in 100 µl PBS/FITC Dextran (40 kDa; Sigma-Aldrich) solution [30 mg/ml] and immediately injected i.v. into a naïve C57Bl/6 recipient. The FITC/Dextran was used for vessel staining. For intravital imaging the animal was anesthetized by a 1% isoflurane inhalation narcosis. Subsequently the skull was thinned carefully with an electric drill and 2-photon microscopy was carried out using a Zeiss LSM 710 NLO microscope on an upright Axio Examiner stage equipped with a 20xNA1.0 water dipping lens (Zeiss, Jena, Germany). For imaging, different areas along the skull window were scanned down to a depth of 400 µm using an illumination wavelength of 850 nm detecting green (514 nm) and red (576 nm) fluorescence with external non descanned detectors (NDD). Time series were recorded at a frame rate of 0.16 fps. During microscopy the mouse was fixed with a stereotactic device and maintained at 37°C by use of an appropriately pre-warmed heating mat.

### Peritoneal neutrophil recruitment

Straight after neutrophil isolation by negative depletion cells were treated with either pertussis toxin (List Biological Laboratories; Campbell, USA) [200 ng/ml] or PBS [10 µl/ml] as control group for 60 min at 37°C. After a subsequent washing step with 10 ml PBS cell staining was carried out using CellTrace™ CFSE (Invitrogen) ([10 nM] 10 min RT) or CellTrace™ Violet (Invitrogen) ([5 µM] 20 min 37°C), one dye for each of the respective pretreated groups. Finally the obtained cell populations were mixed in a 1:1 ratio (1*10^6^ cells type A +1×10^6^ cells type B) and injected i.v. into naïve C57BL/6 recipients in a total volume of 150 µl PBS. 15 min later peritonitis was induced by i.p. injection of 15 ng LPS dissolved in 100 µl PBS. The control group received just 100 µl PBS. Neutrophil inflammation was assessed 2 h later by FACS analysis of blood and peritoneal lavage fluids stained for Gr-1^+^ cells. To exclude effects on cell behavior by the two different tracing dyes all experiments were repeated with a reverse staining.

### Statistical analysis

An unpaired Student *t* test was used to determine the significance of differences between means by calculating two-tailed p-values with a confidence interval of 95%. Calculation was done with the Prism software suite, version 5.01 (Graphpad, La Jolla, USA).

## Supporting Information

Movie S1 **Migration of adoptively transferred neutrophils in vivo.** Neutrophils isolated with the protocol described in this study were labeled with CTO, injected into a C57/BL6 recipient who also received FITC-labelled dextran i.v. Imaging was performed in the region of the cortex after anesthesia and surgical opening of the skull. Shown is an overview of the whole imaging area as well as a close up of a vessel, where red migrating neutrophils can be seen. The real time in seconds is indicated at the lower right.
(MPG)
